# TIPPING POINTS PILOT STUDY: WEARABLE DEVICES AND ALGORITHMS

**DOI:** 10.1093/geroni/igz038.2219

**Published:** 2019-11-08

**Authors:** Rachel L Peterson, Kim Shea, Kayla Luque, Jessica Powell, Jian Liu, Janice crist

**Affiliations:** 1 University of Arizona Center on Aging, Tucson, Arizona, United States; 2 University of Arizona College of Nursing, Tucson, Arizona, United States; 3 University of Arizona College of Engineering, Tucson, Arizona, United States

## Abstract

Little is known about the use and acceptability of fitness watches (e.g. Fitbit) by diverse older adults, or how data from affordable (<$40) devices could be triangulated with self-report data to predict adverse health outcomes. We investigated interest and acceptability of fitness watch technology among Mexican American older adults via a week-long trial. Participants were asked to consistently wear the watches and complete daily diaries of activity, questionnaires, and a semi-structured interview. The watch data was triangulated with data from the daily diaries and questionnaires to validate its usefulness in developing algorithms that could detect important physiological transitions that lead to tipping points. Interview data was qualitatively analyzed and coded for barriers and facilitators of watch use and acceptability. Preliminary results suggest that participants are eager and willing to wear a fitness device. Participants reported interest in monitoring their health and using the device to track and improve physical activity.

